# Diagnostic Value of Mutation-Specific Antibodies for Immunohistochemical Detection of Epidermal Growth Factor Receptor Mutations in Non-Small Cell Lung Cancer: A Meta-Analysis

**DOI:** 10.1371/journal.pone.0105940

**Published:** 2014-09-09

**Authors:** Zi Chen, Hong-bing Liu, Chun-hua Yu, Ying Wang, Li Wang, Yong Song

**Affiliations:** 1 Department of Respiratory Medicine, Jinling Hospital, Nanjing University School of Medicine, Nanjing, P. R. China; 2 Department of medical Engineering, Jinling Hospital, The Second Military Medical University, Nanjing, P. R. China; 3 Department of Respiratory Medicine, Yijishan Hospital, Wannan Medical Collage, Wuhu, P. R. China; University of Algarve, Portugal

## Abstract

**Background:**

Various studies have assessed the diagnostic accuracy of EGFR mutation-specific antibodies in non-small cell lung cancer (NSCLC). We performed a meta-analysis of existing data to investigate the diagnostic value of mutation-specific antibodies for detection of EGFR mutations in NSCLC.

**Methods:**

We systematically retrieved relevant studies from PubMed, Web of Knowledge, and Google Scholar. Data from studies that met the inclusion criteria were extracted for further exploration of heterogeneity, including calculation of the average sensitivity, specificity, positive likelihood ratio (PLR), negative likelihood ratio (NLR), diagnostic odds ratio (DOR), and analysis of SROC(summary receiver operating characteristic) curves.

**Results:**

Fifteen studies met our inclusion criteria. A summary of the meta-analysis of the efficacy of the anti-E746-A750 antibody was as follows: sensitivity, 0.60 (95% CI, 0.55–0.64); specificity, 0.98 (95% CI, 0.97–0.98); PLR, 33.50 (95% CI, 13.96–80.39); NLR, 0.39 (95% CI, 0.30–0.51) and DOR, 111.17 (95% CI, 62.22–198.63). A similar meta-analysis was performed for the anti-L858R antibody with results as follows: sensitivity, 0.76 (95% CI, 0.71–0.79); specificity, 0.96 (95% CI, 0.95–0.97); PLR, 24.42 (95% CI, 11.66–51.17); NLR, 0.22 (95% CI, 0.12–0.39) and DOR, 126.66 (95% CI, 54.60–293.82).

**Conclusion:**

Immunohistochemistry alone is sufficient for the detection of EGFR mutations if the result is positive. Molecular-based analyses are necessary only if the anti-E746-A750 antibody results are negative. Immunohistochemistry seems more suitable for clinical screening for EGFR mutations prior to molecular-based analysis.

## Introduction

Lung cancer is the most frequent cause of cancer-related death worldwide [1]. Non-small cell lung cancer (NSCLC) makes up approximately 80% of lung cancer and is rapidly becoming one of the major diseases that threatens human health. Somatic mutations in the epidermal growth factor receptor (EGFR) gene are found in approximately 10%–16% of NSCLC patients in United States and Europe [2] and 30%–50% of patients in Asia [3]. The two most common genetic mutations are the in-frame deletion in exon 19 (E746-A750) and the substitution of leucine 858 by arginine in the exon 21(L858R) [4]. These two mutations constitute about 90% of all mutations and are known as the “classical” mutations [5]. These two EGFR-specific mutations are strong predictors of the response to small-molecule EGFR-tyrosine kinase inhibitors such as gefitinib [6], [7] and erlotinib [8].

Direct DNA sequencing is a classical method for EGFR mutation detection. However, expensive equipment and amount of time are necessary for this technique. Furthermore, it is difficult to extract the required amounts of high quality DNA from pure tumor cells, which limits direct sequencing in clinical usage. Recently, several other molecular-based analyses have been developed to detect EGFR mutations, including the Scorpion amplification refractory mutation system (ARMS), Smart Amplification Process (SMAP), polymerase chain reaction-single strand conformation polymorphism (PCR-SSCP), and high resolution melting analysis (HRMA), etc. These novel methods require less tumor tissue and less time while achieving high sensitivities and specificities. However, they require advanced operating skills and sophisticated equipment, which hampers their application in clinical practice.

Therefore, it would be beneficial to find an easy, cost-effective, and accurate method to identify EGFR-mutations in NSCLC. Use of immunohistochemistry (IHC) to identify mutant EGFR proteins via specific antibodies is an example of such a method. Yu et al [9] immunized New Zealand rabbits with synthetic peptides matching the EGFR sequence with the E746-A750 deletion in exon 19 or the L858R point mutation in exon 21. By contrast, conflicting results are reported by several recent studies on the potential diagnostic value of mutation-specific antibodies for immunohistochemical detection of EGFR mutations in NSCLC. For instance, the sensitivity of anti-E746-A750 antibody was 36% reported by Hofman et al [10] while it reached 100% in Hasanovic et al study [11].

In order to clarify the value of mutation-specific antibodies in the identification of EGFR mutation status, a meta-analysis was conducted to systematically and quantitatively evaluate the accuracy of the immunohistochemical method for EGFR mutation screening in NSCLC.

## Material and Methods

### Data sources and searches

We identified relevant studies by searching PubMed, Web of Knowledge, and Google Scholar. We limited our search to English language literature published between May 2009 and July 2013. The keywords used included ‘immunohistochemistry’, ‘EGFR mutation’, ‘NSCLC’, ‘non-small cell lung cancer’, ‘lung carcinoma’, ‘lung adenocarcinoma’, ‘pulmonary adenocarcinoma’, and ‘mutation-specific antibodies’. Articles were also identified by use of the related articles function in PubMed.

Two reviewers (Zi Chen and Hong-bing Liu) inspected the title and abstract of each citation independently to identify those studies that were likely to report the diagnostic value of EGFR mutation-specific antibodies. For those articles that were not excluded based on title and abstract, reviewers retrieved full text, made judgment and decided final conclusion for them. If disagreement occurred, two reviewers discussed and arrived at consensus (Zi Chen and Hong-bing Liu). Inclusion criteria for the primary studies were as follows: (1) all samples were NSCLC, confirmed either histologically or cytologically; (2) must have used the authoritative molecule-based standard for the EGFR mutation and immunohistochemical staining score criteria. (3) results in each individual study could be summarized in a 2×2 contingency table; and (4) there were no restrictions as to data collection timing (i.e., prospective or retrospective).

Review articles, editorials, case reports, and corresponding letters were excluded due to lack of original data. If we found multiple articles for a single study, we used the best-quality one.

### Data extraction and quality assessment

Two investigators (Zi Chen and Hong-bing Liu) extracted the following data from independently the selected studies: (1) year of publication; (2) location of the study; (3) number of tumor tissue or cytology specimens; (4) IHC methodology; (5) IHC score criteria; (6) standard; (7) number of true positive (TP); (8) number of false positive (FP); (9) number of false negative (FN), and (10) number of true negative (TN) for the exon 19 deletion and exon 21 L858R mutation, respectively. In addition, for an accurate evaluation of heterogeneity, the following characteristics of study design were retrieved: (1) whether the study was double-blind regarding the results of the immunohistochemical method and the results of the molecule-based analysis; (2) whether there was consecutive or random sampling of patients; and (3) tissue sample preparation [whether FFPE (Formalin-Fixed Paraffin-Embedded) was used]. The Quality Assessment of Diagnostic Accuracy Studies (QUADAS, maximum score 14) [12] and the Standards for Reporting Diagnostic accuracy (STARD, maximum score 25) [13] were used to assess the quality of the selected studies. Disagreements were resolved by discussion between Zi Chen and Hong-bing Liu.

### Statistical analysis

We used standard methods recommended for meta-analysis of diagnostic test evaluations [14]. Firstly, we tested for the presence of cut-off point effects. Estimates of diagnostic accuracy differ if not all studies use the same cut-off point for a positive test result or for the reference standard. Variation in the parameters of accuracy may be partly due to variation in cut-off point. We tested for the presence of a cut-off point effect between studies by calculating a Spearman correlation coefficient between sensitivity and specificity of all included studies [14]. A positive rank-correlation coefficient and a p<0.05 are suggestive of a significant cut-off point effect. If the cut-off point effect was present, the sensitivity, specificity, LR and DOR of each research were not suitable for merger.

A SROC curve was the basis of the meta-analysis [14], [15]. The SROC curve was plotted to identify the sensitivity and specificity for the single test threshold from each study [15], [16]. We calculated the respective area under SROC curve and Q* index on SROC curve where sensitivity equals to specificity. A random-effects model (REM) was used to calculate the average sensitivity, specificity, positive likelihood ratio (PLR), negative likelihood ratio (NLR) and diagnostic odds ratio (DOR) [17].

The DOR is a common comprehensive evaluation indicator, which combines data from sensitivity, specificity, PLR and NLR into a single number: (TP/FN)/(FP/TN) [18]. The DOR of a test is the ratio of the odds of positive test results in NSCLC patients with EGFR mutations relative to the odds of positive test results in the wild-type patients. The value of a DOR ranges from 0 to infinity, with higher values implying better discriminative test performance.

In this meta-analysis, besides cut-off point effect, there were additional factors that can cause heterogeneity as well. Majority diagnostic reviews indicate considerable heterogeneity in the results of included studies [14]. When different studies have largely different results, this may result from either random error or heterogeneity due to differences in clinical or methodological characteristics of studies [14]. We used the I^2^ test for the pooled DOR (PDOR) to detect statistically significant heterogeneity [19]. The PDOR was computed according to standard methods to analyze the changing in diagnostic accuracy in the study per unit in the covariates [20]. I^2^≥50% indicated substantial heterogeneity. We included the STARD and the QUADAS as covariates in univariate meta-regression analysis by assessing the effects of their score on the diagnostic ability of mutation-specific antibodies. We also analyzed the effects of other covariates on blinded design, consecutive or random specimen of patients, IHC methodology, IHC score criteria, and standard. Subgroup analysis was performed to explore the sources of potential heterogeneity among studies using univariate meta-regression analysis. As publication bias is of concern for the meta-analyses of diagnostic studies, we tested for the potential presence of this bias using Deeks' funnel plots. [21]

All analyses were performed using two statistical software programs, Stata, version 12.0 (Stata Corporation, College Station, TX, USA) and Meta-Disc 1.4 for Windows (XI Cochrane Colloquium, Barcelona, Spain). The statistical significance was set at p<0.001.

## Results

### Eligible studies and quality assessment

**Figure 1:**
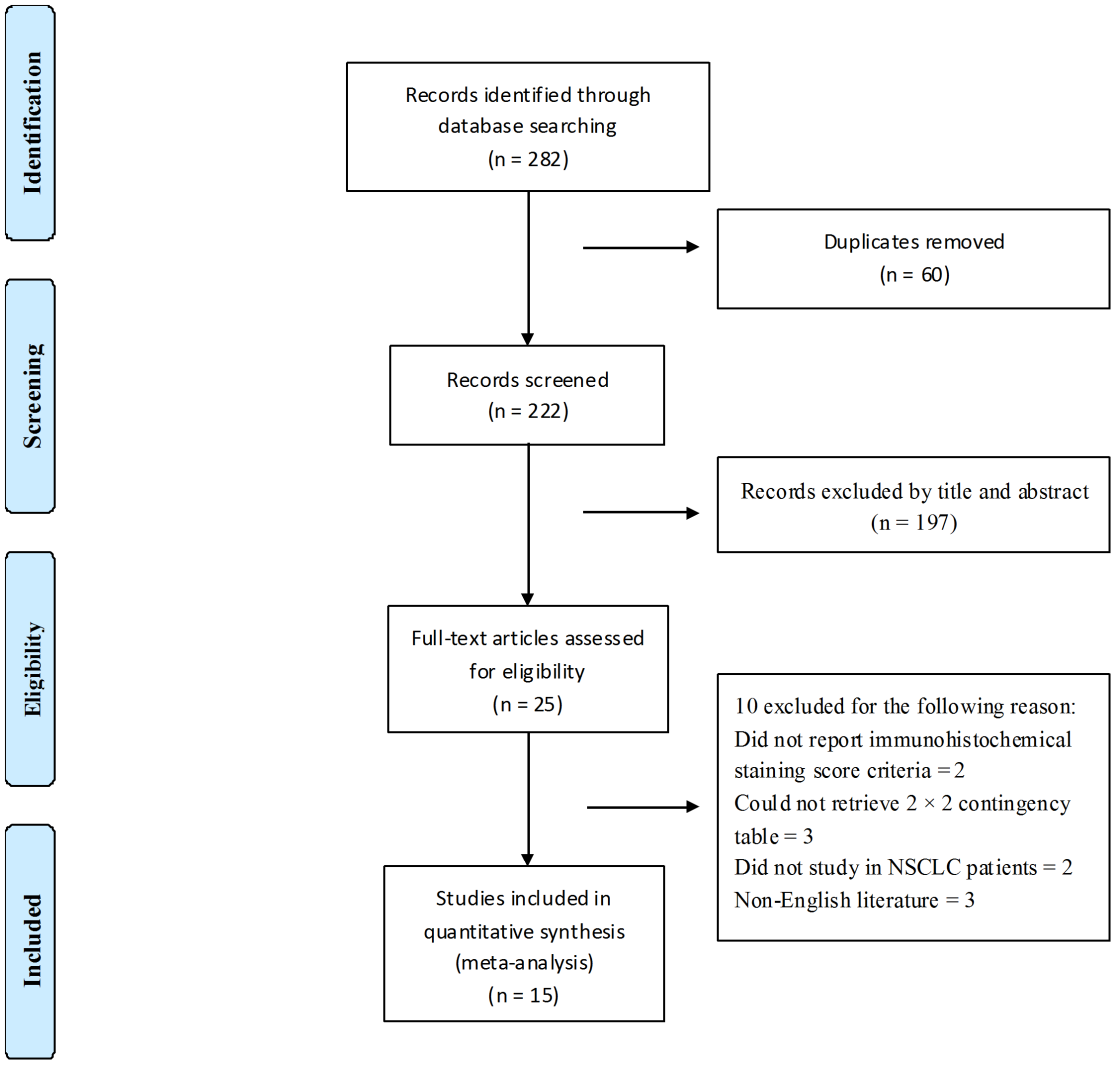
Flow diagram of study selection by using electronic database.

As shown in figure 1, the literature search identified fifteen published studies[9]–[11], [22]–[33] that fulfilled the inclusion criteria. Summaries and characteristics of these studies are reported in table 1–3. Overall, 2337 cases were entered in the meta-analysis, ranging from 33 to 577 patient specimens per study. As shown in table 1 and table 2, seven of the fifteen studies (47%) used tissue microarray technology in the immunohistochemical method; twelve studies (80%) set the four grades of visual scoring as the IHC score criteria; in twelve studies (80%) direct sequencing was used as the standard. As shown in the table 3, three of fifteen studies (20%) used a double-blind study design while evaluating the accuracy of detecting EGFR mutation status by molecular-based analyses compared with the immunohistochemical method. In twelve studies (80%), the specimens were collected from consecutive or randomized patients. The NSCLC was confirmed by histological and cytological examination. The characteristics, together with the STARD and the QUADAS scores of these studies are outlined in table 3.

**Table 1:** Summary of included studies.

Reference/year	Country/region	No.	IHC methodology	IHC score criteria	Exon 19 deletion				Exon 21 L858R				Standard
					TP	FP	FN	TN	TP	FP	FN	TN	
Yu et al (2009) [9]	USA	244	TMA	4 grades, visual scoring^a^	23	0	3	196	24	2	2	193	Direct sequencing
Brevet et al (2010) [24]	USA	194	TMA	4 grades, visual scoring^a^	23	2	8	161	20	2	1	171	PCR-RFLP and sequenom assays
Simonetti et al (2010) [31]	Spain	78	Individual slides	4 grades, visual scoring^b^	20	0	9	49	25	0	2	51	Fragment analysis, TaqMan assay, and direct sequencing
Kawahara et al (2010) [28]	Japan	60	Individual slides	4 grades, visual scoring^b^	13	0	8	39	19	1	4	36	Direct sequencing
Kitamura et al (2010) [29]	Japan	238	TMA	4 grades, visual scoring^a^	16	1	25	196	12	6	25	195	Direct sequencing, Cycleave PCR, and fragment analysis
Ilie et al (2010) [26]	France	61	TMA	4 grades, visual scoring^a^	9	4	1	47	0	0	0	0	Direct sequencing
Kozu et al (2010) [30]	Japan	577	TMA	Q score criteria^c^	57	2	78	440	130	9	42	396	HRMA

a The visual score criteria consider 1+ or more staining as positive.

b The visual score criteria consider 2+ or 3+ staining as positive.

c Q score was calculated by multiplying the percentage (P, 0–100%) of positive cells by the intensity (I, 0–3) of staining (Q = P*I; maximum  = 300) [30].

Abbreviations: IHC, immunohistochemistry; TMA, tissue microarray; AUC, the best area under the ROC curves; TP, true positive; FP, false positive; FN, false negative; TN, true negative; PCR-RFLP, Polymerase chain reaction-restriction fragment length polymorphism; HRMA, high resolution melting analysis.

**Table 2:** Summary of included studies.

Reference/year	Country/region	No.	IHC methodology	IHC score criteria	Exon 19 deletion				Exon 21 L858R				Standard
					TP	FP	FN	TN	TP	FP	FN	TN	
Kato et al (2010) [27]	Japan	70	TMA	H score criteria^d^	9	0	9	52	9	2	3	56	Direct sequencing
Hofman et al (2012) [10]	France	151	Individual slides	4 grades, visual scoring^a^	13	3	23	111	4	2	17	122	Direct sequencing
Wu et al (2011) [32]	Chinese Taipei	143	Individual slides	Q score criteria^c^	30	5	11	97	38	23	5	77	Direct sequencing
Hasanovic et al (2012) [11]	USA	145	Individual slides	4 grades, visual scoring^b^	41	24	0	80	29	15	0	101	PCR-RFLP and sequenom assays
Ambrosini-Spaltro et al (2012) [22]	Italy	33	Individual slides	4 grades, visual scoring^b^	6	0	6	21	5	0	1	27	Direct sequencing
Angulo et al (2012) [23]	Spain	89	Individual slides	4 grades, visual scoring^b^	8	0	3	78	2	0	3	78	Direct sequencing
Cooper et al (2013) [25]	Austria	204	TMA	4 grades, visual scoring^b^	7	4	0	193	6	3	1	194	Direct sequencing
Xiong et al (2013) [33]	China	50	Individual slides	4 grades, visual scoring^b^	10	0	7	33	13	1	3	33	Mutant-enriched PCR and direct sequencing

a The visual score criteria consider 1+ or more staining as positive.

b The visual score criteria consider 2+ or 3+ staining as positive.

c Q score was calculated by multiplying the percentage (P, 0–100%) of positive cells by the intensity (I, 0–3) of staining (Q = P*I; maximum  = 300) [30].

d H score criteria assessed staining intensity (I, 0–4) multiplied by the percentage (P, 0–100%) of positive cells for each intensity for a final IHC score (H = P*I; maximum  = 400) [27].

Abbreviations: IHC, immunohistochemistry; TMA, tissue microarray; AUC, the best area under the ROC curves; TP, true positive; FP, false positive; FN, false negative; TN, true negative; PCR-RFLP, Polymerase chain reaction-restriction fragment length polymorphism; HRMA, high resolution melting analysis.

**Table 3:** Characteristics of included studies.

Reference/year	Blinded design	Consecutive or random	Tissue sample preparation	Quality score	
				STARD	QUADAS
Yu et al (2009) [9]	Yes	Unknown	FFPE	10	11
Brevet et al (2010) [24]	Unknown	Yes	FFPE	13	11
Simonetti et al (2010) [31]	No	Unknown	FFPE	10	12
Kawahara et al (2010) [28]	Unknown	Yes	FFPE	12	11
Kitamura et al (2010) [29]	No	Yes	FFPE/frozen	12	11
Ilie et al (2010) [26]	Unknown	Yes	FFPE/frozen	14	10
Kozu et al (2010) [30]	Unknown	Yes	MFPE/frozen	15	12
Kato et al (2010) [27]	Unknown	Unknown	FFPE	15	12
Hofman et al (2012) [10]	Yes	Yes	FFPE	12	10
Wu et al (2011) [32]	Unknown	Yes	FFPE	19	12
Hasanovic(2012) [11]	Unknown	Yes	FFPE	13	8
Ambrosini-Spaltro et al (2012) [22]	Unknown	Yes	FFPE	9	12
Angulo et al (2012) [23]	Unknown	Yes	FFPE	12	10
Cooper et al (2013) [25]	Yes	Yes	FFPE	17	13
Xiong et al (2013) [33]	Unknown	Yes	FFPE	17	11

Abbreviations: FFPE, formalin-fixed paraffin-embedded; MFPE, methanol-fixed paraffin-embedded; STARD, the Standards for Reporting Diagnostic accuracy, QUADAS, the Quality Assessment of Diagnostic Accuracy Studies.

### Heterogeneity assessment and diagnostic accuracy

The Spearman correlation coefficient of the anti-E746-A750 antibody and the anti-L858R antibody were 0.360 (P = 0.187) and −0.033 (P = 0.911), respectively, verifying that the variability across these studies could not be explained by differences in the diagnostic cut-off point (since the P-values were not <0.05).

**Figure 2:**
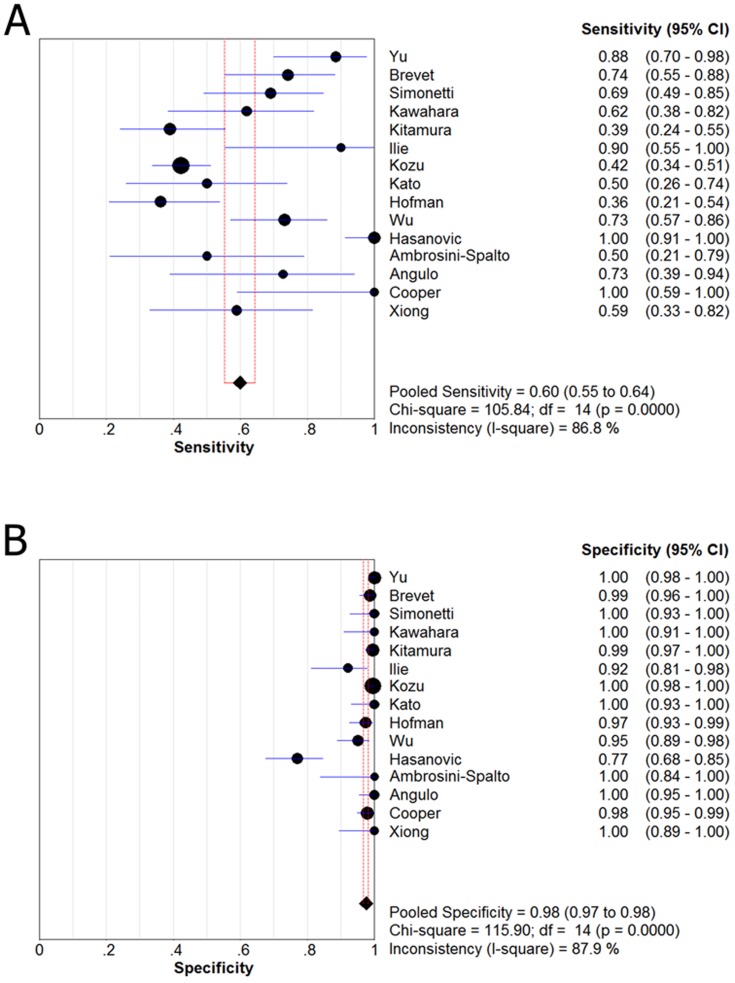
Forest plots for sensitivity (A) and specificity (B) of the anti-E746-A750 antibody in the detecting the EGFR exon 19 deletion. Sensitivity  = 0.60 (95% CI, 0.55–0.64); specificity  = 0.98 (95% CI, 0.97–0.98).

**Figure 3:**
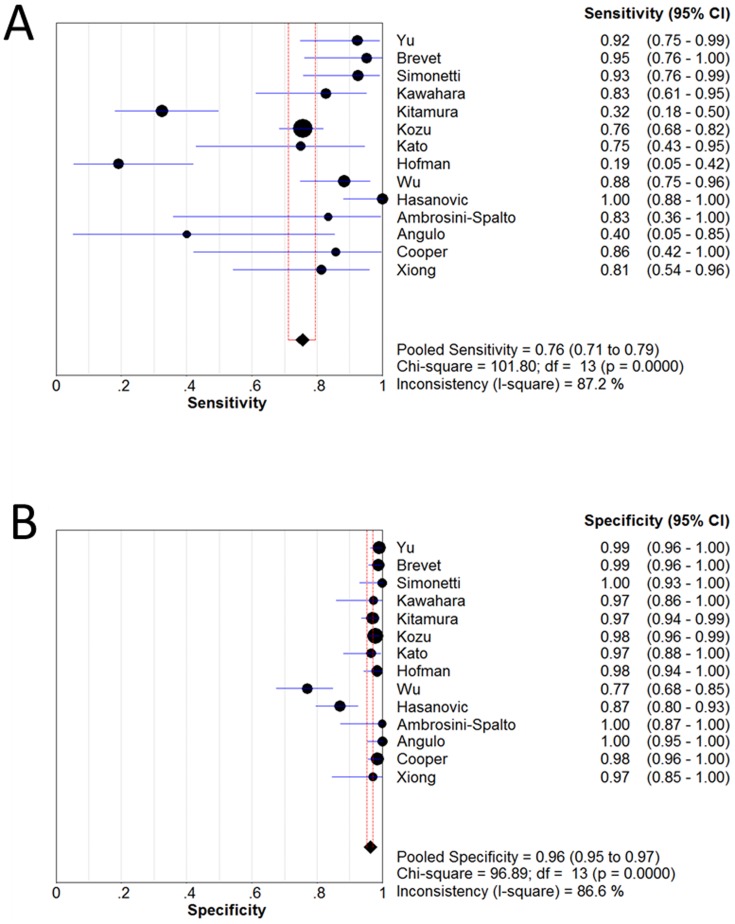
Forest plots for sensitivity (C) and specificity (D) of the anti-L858R antibody in the detecting the EGFR exon 21 mutation. Sensitivity  = 0.76 (95% CI, 0.71–0.79); specificity  = 0.96 (95% CI, 0.95–0.97).

**Figure 4:**
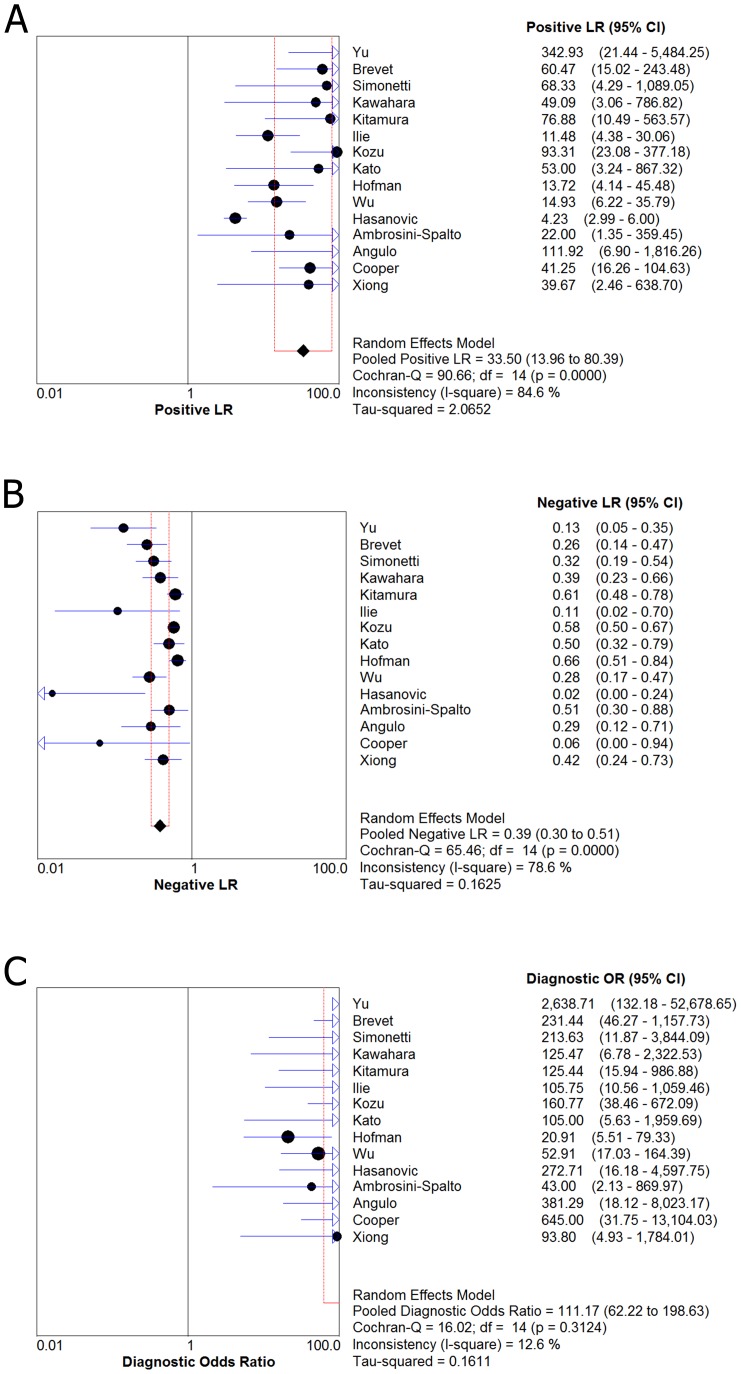
Forest plot for the positive likelihood ratio (PLR) (A), the negative likelihood ratio (NLR) (B) and the diagnostic odds ratio (DOR) (C) of the anti-E746-A750 antibody. PLR (positive likelihood ratio)  = 33.50 (95% CI, 13.96–80.39); NLR (negative likelihood ratio)  = 0.39 (95% CI, 0.30–0.51); DOR (diagnostic odds ratio)  = 111.17 (95% CI, 62.22–198.63).

**Figure 5:**
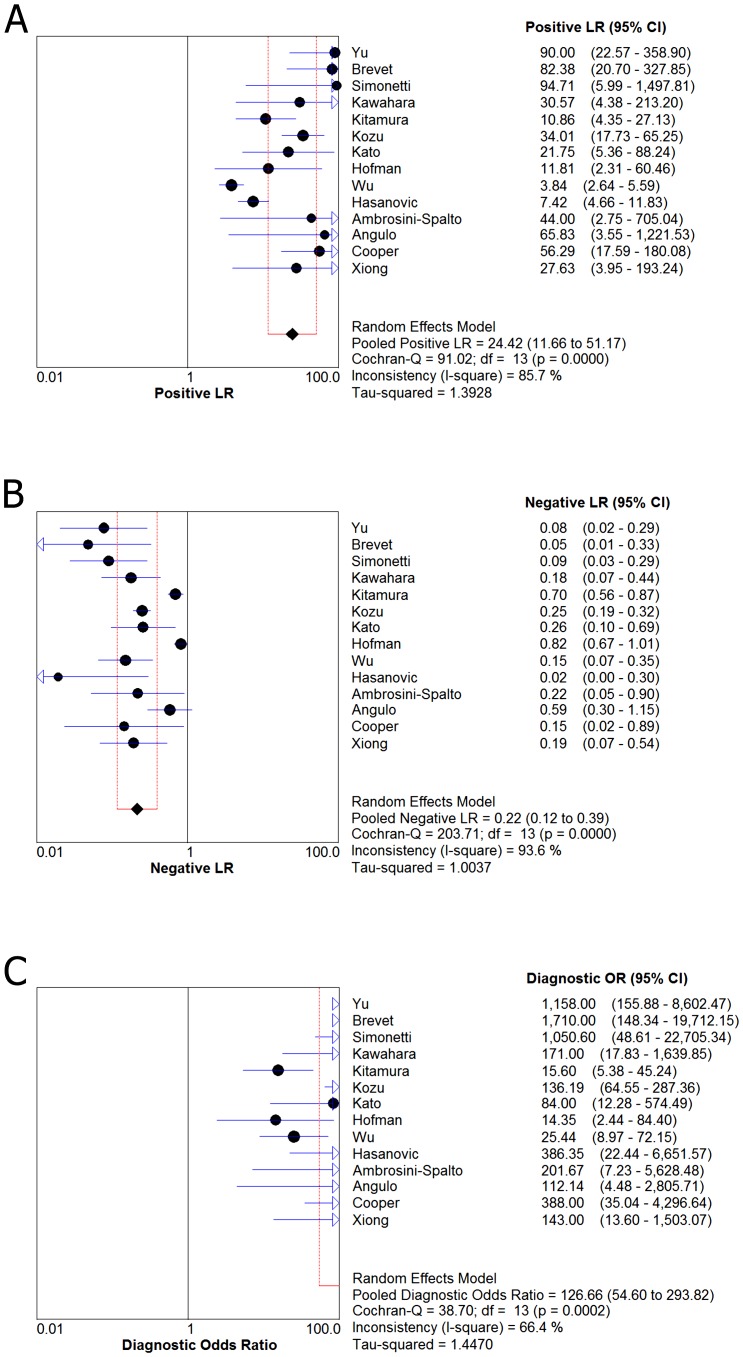
Forest plot for the positive likelihood ratio (PLR) (A), the negative likelihood ratio (NLR) (B) and the diagnostic odds ratio (DOR) (C) of the anti-L858R antibody. PLR (positive likelihood ratio)  = 24.42 (95% CI, 11.66–51.17); NLR (negative likelihood ratio)  = 0.22 (95% CI, 0.12–0.39); DOR (diagnostic odds ratio)  = 126.66 (95% CI, 54.60–293.82).

Figure 2 and 3 shows forest plots of sensitivity and specificity for the anti-E746-A750 antibody and the anti-L858R antibody in the identification of EGFR mutation status. For theanti-E746-A750 antibody, the sensitivity ranged from 0.36 to 1.00 (mean, 0.60; 95% confidence interval (CI), 0.55–0.64), while specificity ranged from 0.77 to 1.0 (mean, 0.98; 95% CI, 0.97–0.98). For the anti-L858R antibody, the sensitivity ranged from 0.19 to 1.00 (mean, 0.76; 95% CI), 0.71–0.79), while specificity ranged from 0.77 to 1.0 (mean, 0.96; 95% CI, 0.95–0.97). In figure 4, we also noted that the PLR, NLR, and DOR of the anti-E746-A750 antibody were 33.50 (95% CI, 13.96–80.39), 0.39 (95% CI, 0.30–0.51), and 111.17 (95% CI, 62.22–198.63), respectively; the PLR, NLR, and DOR of the anti-L858R antibody were 24.42 (95% CI, 11.66–51.17), 0.22 (95% CI, 0.12–0.39), and 126.66 (95% CI, 54.60–293.82), respectively (figure 5). For the anti-E746-A750 antibody, the I^2^ test for PDOR was 12.6%, which did not show any major qualitative evidence for heterogeneity between studies. Regarding PLR and NLR, we found significant heterogeneity for all of the inclusion studies, I^2^ = 84.6% and 78.6%, respectively. For the anti-L858R antibody, the I^2^ test for PDOR, PLR, and NLR were 66.4%, 85.7%, and 93.6%, respectively, which showed substantial heterogeneity among studies.

**Figure 6:**
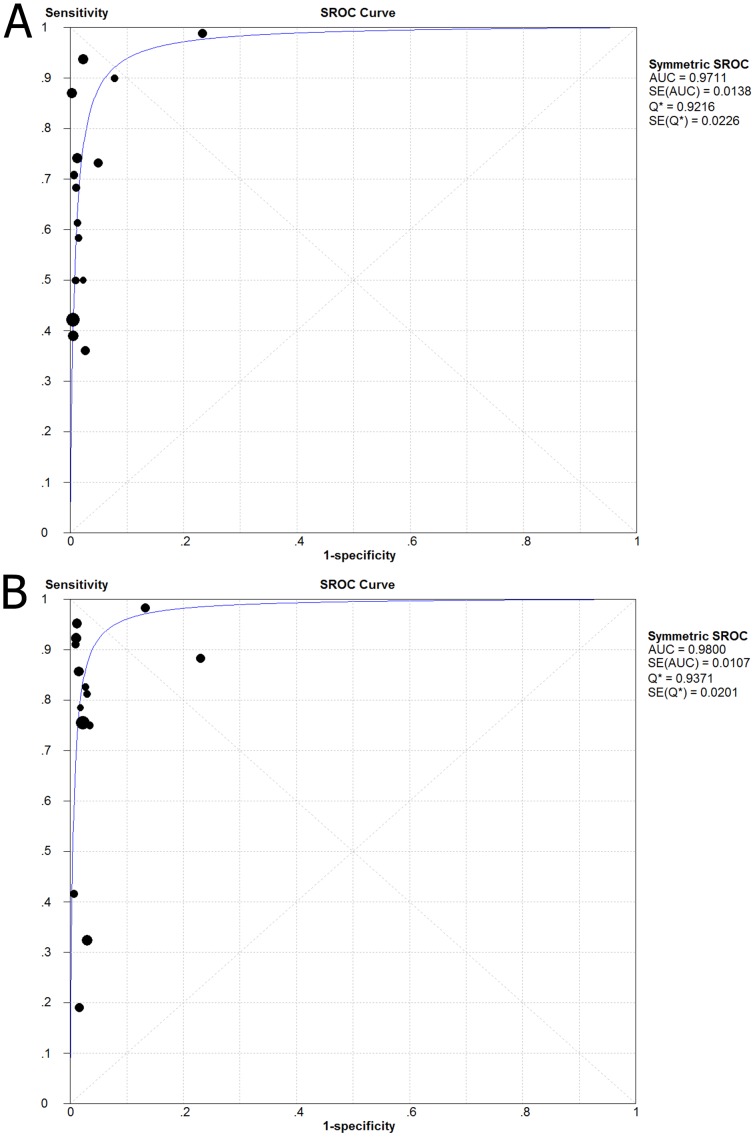
Summary receiver operating characteristic (SROC) curve for the anti-E746-A750 antibody (A) and the anti-L858R antibody (B) in the diagnosis of EGFR mutation in the 15 included studies. The anti-E746-A750 antibody: AUC = 0.97, Q* = 0.92; the anti-L858R antibody: AUC = 0.98, Q* = 0.94.

Besides indicating the degree of equivalency between sensitivity and specificity, the SROC curve and the area under the curve also give a general summary of performance. Graphs of the SROC curves for the anti-E746-A750 antibody and the anti-L858R antibody the rate of true-positives compared with the rate of false-positives from individual studies are shown in figure 6. For the anti-E746-A750 antibody, the area under the curve (AUC) was 0.9711 (Q* index  = 0.9216); for the anti-L858R antibody, the area under the curve (AUC) was 0.9800 (Q* index  = 0.9371). These data indicate that both mutation-specific antibodies represent a high level overall accuracy.

### Meta regression and sub-group analysis

A quality score for every study was completed using STARD guidelines [13], in which each score is compiled on the basis of title and introduction, methods, results and discussion (table 3). The QUADAS tool [12] was also used to scale the score by 1, when a criterion was fulfilled; 0, if a criterion was unclear and −1, if the criterion was not achieved (table 3). These scores were employed in the meta-regression analysis to evaluate the effect of study quality on the PDOR of mutation-specific antibodies in identification of the EGFR mutation status.

For the anti-E746-A750 antibody, as shown in table 4, studies with lower quality (STARD score<13; QUADAS score<10) had PDOR values that were not obviously lower than those of studies of higher quality. We also noted that differences among studies with or without blinded design, consecutive or random design, IHC methodology [tissue microarray (TMA) vs. individual slides], IHC score criteria (consider 2+ or 3+ as positive vs. others), and standard used for immunohistochemical method (direct sequencing vs. others) did not reach statistical significance, which implies that the diagnostic accuracy was not substantially affected by the design of study.

**Table 4:** Weighted meta-regression analysis of the effects of methodological and study design on diagnostic value of mutation-specific antibodies.

Covariates	No.	Coefficient	PDOR(95% CI)	*p* value
**Anti-E746-A750 antibody**	15			
STARD score ≥13	8	−1.168	0.31 (0.02;4.88)	0.3392
QUADAS score ≥10	13	0.159	1.17 (0.00;1108.68)	0.9565
Blinded	3	0.610	1.84 (0.22;15.58)	0.5105
Consecutive or random	12	−1.204	0.30 (0.03;3.43)	0.2721
IHC methodology	6	2.488	12.04 (0.87;166.99)	0.0598
IHC score criteria	8	2.209	9.11 (0.88;93.87)	0.0597
Standard	12	−1.437	0.24 (0.02;2.89)	0.2087
**Anti-L858R antibody**	14			
STARD score ≥13	7	−1.137	0.32 (0.03;3.11)	0.2546
QUADAS score ≥10	12	2.879	17.81 (0.11;2840.99)	0.2043
Blinded	3	1.223	3.40 (0.52;22.38)	0.1564
Consecutive or random	11	−2.161	0.12 (0.02;0.78)	0.0333
IHC methodology	6	0.680	1.97 (0.20;19.54)	0.4805
IHC score criteria	7	2.704	14.94 (1.61;138.51)	0.0262
Standard	11	−2.992	0.05 (0.01;0.34)	0.0102

Abbreviations: PDOR, pooled diagnostic odds ratio; CI, confidence interval; STARD, the Standards for Reporting Diagnostic accuracy; QUADAS, the Quality Assessment of Diagnostic Accuracy Studies; IHC, immunohistochemistry.

For the anti-L858R antibody, as shown in table 4, we noticed that differences quality, blinded design, and IHC methodology among studies did not contribute to the heterogeneity. However, the differences among consecutive or random design, IHC score criteria, and standard reached statistical significance, which indicates that the study design could affect the diagnostic accuracy.

According to the results of meta-regression, we performed a sub-group analysis, and the data was show in table 5 and table 6.

**Table 5:** Potential sources of heterogeneity of sub-group analysis.

Studies		Sensitivity(95%CI)	Specificity(95%CI)	PLR (95% CI)*	NLR (95% CI)*	DOR (95% CI)*	AUC
**E746-A750**							
All studies (15)		0.60(0.55–0.64)	0.98(0.97–0.98)	33.50(13.96–80.39)	0.39(0.30–0.51)	111.7(62.22–198.63)	0.9711
I-square (%)		86.8	87.9	84.6	78.6	12.6	
Consecutive or random	Yes (12)	0.58(0.53–0.63)	0.97(0.96–0.98)	26.99(11.03–66.06)	0.41(0.31–0.55)	89.92(51.94–155.65)	0.9686
	I-square (%)	88.2	89.0	85.0	77.1	0.1	
	No or Unknown (3)	0.71(0.59–0.81)	1.00(0.99–1.00)	107.87(21.69–536.52)	0.30(0.15–0.64)	379.59(55.57–2592.76)	0.9995
	I-square (%)	75.4	0.0	0.0	77.3	22.2	
IHC score criteria	Consider 2+ or 3+ staining as positive (7)	0.76(0.68–0.83)	0.95(0.92–0.96)	29.05(6.68–126.31)	0.34(0.21–0.54)	184.15(60.47–560.81)	0.9818
	I-square (%)	83.1	91.0	84.3	64.0	0.0	
	Others (8)	0.53(0.48–0.59)	0.99(0.98–0.99)	33.47(15.04–74.44)	0.43(0.32–0.58)	104.21(44.93–241.69)	0.9529
	I-square (%)	85.5	73.3	59.5	80.7	44.2	
Standard	Direct sequencing (12)	0.61(0.55–0.67)	0.98(0.98–0.99)	26.86(15.76–45.76)	0.39(0.29–0.53)	95.07(46.06–196.22)	0.9657
	I-square (%)	74.9	62.1	21.1	73.4	18.8	
	non-Direct sequencing (3)	0.58(0.51–0.65)	0.96(0.94–0.97)	27.48(1.50–503.58)	0.25(0.07–0.89)	197.71(72.73–537.46)	0.9817
	I-square (%)	96.8	97.4	95.8	91.2	0.0	

Abbreviations: CI, confidence interval; PLR, positive likelihood ratio; NLR, negative likelihood ratio; DOR, diagnostic odds ratio; AUC, area under the receiver operating characteristic curve; IHC, immunohistochemistry.

*PLR (95% CI), NLR (95% CI), and DOR (95% CI) was calculated using random effect model.

**Table 6:** Potential sources of heterogeneity of sub-group analysis.

Studies		Sensitivity(95%CI)	Specificity(95%CI)	PLR (95% CI)*	NLR (95% CI)*	DOR (95% CI)*	AUC
**L858R**							
All studies (14)		0.76(0.71–0.79)	0.96(0.95–097)	24.42(11.66–51.17)	0.22(0.12–0.39)	126.66(54.60–293.82)	0.9800
I-square (%)		87.2	86.6	85.7	93.6	66.4	
Consecutive or random	Yes (11)	0.73(0.68–0.78)	0.96(0.95–0.97)	20.13(9.14–44.35)	0.25(0.13–0.48)	92.66(37.73–227.53)	0.9741
	I-square (%)	88.9	88.5	86.4	94.0	65.3	
	No or Unknown (3)	0.89(0.79–0.96)	0.99(0.97–1.00)	49.32(17.11–142.18)	0.13(0.06–0.30)	407.34(64.38–2577.22)	0.9995
	I-square (%)	21.9	30.9	18.5	35.0	49.6	
IHC score criteria	Consider 2+ or 3+ staining as positive (7)	0.88(0.80–0.93)	0.96(0.94–0.98)	30.11(9.92–91.40)	0.18(0.08–0.40)	252.55(91.27–698.81)	0.9852
	I-square (%)	62.1	81.5	69.5	69.2	0.0	
	Others (7)	0.71(0.66–0.76)	0.96(0.95–0.97)	21.25(6.63–68.12)	0.26(0.12–0.56)	85.05(26.08–277.37)	0.9768
	I-square (%)	91.7	90.7	91.5	96.0	80.9	
Standard	Direct sequencing (11)	0.70(0.64–0.76)	0.96(0.95–0.97)	25.01(9.60–65.14)	0.26(0.13–0.49)	95.54(35.98–253.71)	0.9733
	I-square (%)	86.9	86.2	82.5	92.4	63.3	
	non- Direct sequencing (3)	0.81(0.75–0.86)	0.96(0.95–0.98)	25.25(5.50–115.80)	0.08(0.01–0.54)	316.51(65.61–1526.79)	0.9882
	I-square (%)	89.5	91.8	92.3	75.3	50.8	

Abbreviations: CI, confidence interval; PLR, positive likelihood ratio; NLR, negative likelihood ratio; DOR, diagnostic odds ratio; AUC, area under the receiver operating characteristic curve; IHC, immunohistochemistry.

*PLR (95% CI), NLR (95% CI), and DOR (95% CI) was calculated using random effect model.

### Evaluation of publication bias

**Figure 7:**
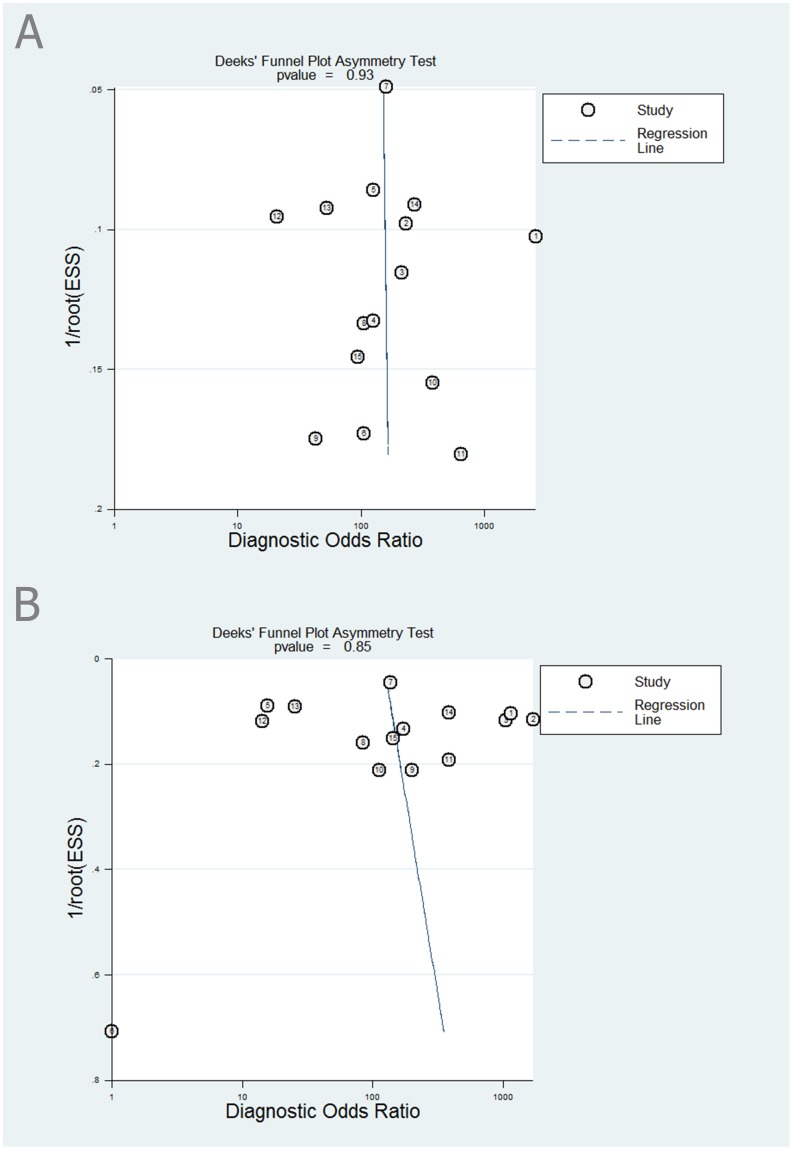
The funnel plot of publication bias for the anti-E746-A750 antibody (A) and the anti-L858R antibody (B). There was no significant publication bias (the anti-E746-A750 antibody, P = 0.93; the anti-L858R antibody, P = 0.85).

The Deek's funnel plot asymmetry test revealed the lack of publication bias (the anti-E746-A750 antibody, P = 0.93; the anti-L858R antibody, P = 0.85) (figure 7) [21].

## Discussion

In recent years, two novel antibodies for IHC have been developed against the most common EGFR mutations, the 15 bp exon 19 deletions and the L858R mutation in exon 21 [9]. The discovery of mutation-specific antibodies opened up a new possibility for the detection of the EGFR mutation in NSCLC. Many studies have been done in order to evaluate its diagnostic power of these antibodies; however, there has been no consensus as to their efficacy. Therefore, in the present study, we analyzed the data by meta-analysis to obtain an accurate conclusion.

The present meta-analysis demonstrates that the L858R antibody has higher sensitivity than the E746-A750 antibody (76% vs. 60%). Considering the sensitivity of the anti-E746-A750 antibody is only 60%, it will increase the risk of a false negative if the pathology technician uses immunohistochemical methods as the sole means of detecting the EGFR exon 19 deletion. As the anti-E746-A750 antibody specifically detects 15-bp deletions, it naturally shows extremely high sensitivity and specificity in 15-bp deletion cases. However, the 15-bp exon 19 deletion mutants account for only 68.1% of the exon 19 deletions in the COSMIC database. Apart from the 15 bp deletions, other exon 19 deletions of sizes 9, 12, 18, or 24-bp occur in NSCLC resulting in slightly different epitopes with deletions of 3–8 amino acids. For non-15-bp exon 19 deletion mutants, the sensitivity varied depending on the deletion size, ranging from 20% to 67% [24]. Originally, Yu et al. reported IHC results on only two non-15-bp deletion cases, of which one was positive by IHC [9]. In Kato et al, all of the exon 19 deletion samples contained seven non-15-bp deletion cases, none of which were positive using the antibody [27]. However, in the chosen 15 studies, the L858R mutation was found in the vast majority of exon 21 mutations, which resulted in a moderately high sensitivity. However, based on our high specificity (the anti-E746-A750 antibody: 99% vs. the anti-L858R antibody: 98%), a positive result could eliminate the need for confirmatory molecular testing.

From the Fig. 2A and 3A, we also found there is a large variation in sensitivity for the two antibodies among the 15 chosen studies. We considered this was due to a limitation of IHC to EGFR mutation testing that only uses mutation-specific antibodies for the commoner EGFR mutations. Therefore, rarer sensitizing mutations in EGFR couldn't be identified. The two most frequent mutations in EGFR in NSCLC are the L858R point mutation in exon 21, And the proportion of these two types of mutation ranged from 52% [24] to 96% [9] of all identified mutations in exon 19 and 21among 15 chosen studies Thus, the higher proportion of common mutations, the higher sensitivity the mutation specific antibodies would be. Kato et al found the overall sensitivity of mutation-specific antibodies for detecting EGFR mutations to be fairly low (43.9%) when all EGFR mutations were taken into account [26]. This result implies the two antibodies are inadequate at detecting variant exon 19 deletions and exon 21 point mutations. Further refinement of these mutation-specific antibodies will be required to cover these rare mutations and to improve the affinity of these antibodies to the antigen. An antibody cocktail could also be developed to detect the common, the rare exon 19 deletions, exon 21 mutations as well as the resistance mutation T790M in exon 20.

The SROC curve and its AUC do not depend on the diagnostic threshold and. In a high quality diagnostic study, the AUC value is close to 1; however, in low quality studies, the AUC value is close to 0.5. The AUC displays a general summary of best performance and reveals the equivalency between sensitivity and specificity. In our meta-analysis, the maximum joint sensitivity and specificity (Q* index) for the anti-E746-A750 antibody is 0.9216 while the AUC is 0.9711; for the anti-L858R antibody, the Q* index is 0.9371 while the AUC is 0.9800. Therefore, the diagnostic accuracy of quantitative analysis of mutation-specific antibodies with immunohistochemical detection of EGFR mutations is similarly effective to molecular-based analyses.

Compared to the SROC curve, which is not easy to interpret and use [34], likelihood ratios are considered to be a more meaningful method in clinical practice [35]; therefore, we also calculated both PLR and NLR as our detections of diagnostic value. In our meta-analysis, the PLR refers to the ratio of the probability of mutation-positive results in EGFR mutant-type patients (true positive rate, TPR) to the probability of mutation-positive results in EGFR wild-type patients (false positive rate, FPR). The PLR indicates the probability of positive test result as compared to EGFR wild-type patients with the immunohistochemical method; a larger ratio indicates a higher diagnostic value of result. The NLR represents the ratio of the probability of mutation-negative results in EGFR mutant-type patients (false negative rate, FNR) to the probability of mutation-negative results in EGFR wild-type patients (true negative rate, TNR). Unlike the PLR, the NLR indicates the probability of a negative test result as compared with EGFR wild-type patients with the immunohistochemical method; therefore, a smaller ratio represents a higher diagnostic value of the result. For the anti-E746-A750 antibody, a PLR value of 33.50 suggests that NSCLC patients with EGFR mutations have about 34-fold higher chance of being IHC-positive compared with wild-type patients. This probability strongly confirms the diagnosis of EGFR mutation status. For the anti-L858R antibody, the PLR value is 24.42; this probability is also high enough to confirm the diagnosis of EGFR mutation status. By contrast, the NLR of the anti-E746-A750 antibody and the anti-L858R antibody were found as 0.39 and 0.22 in the present meta-analysis, respectively. If the immunohistochemical result was negative, the probability that wild-type patients have mutation status is 39% and 22%, respectively. These data illustrate that a negative immunohistochemical result should not be used alone as a justification to deny mutation status in exon 19; however, for the anti-L858R antibody, the result was barely satisfactory. These data indicate that for mutations in both in exon 19 and exon 21, if the immunohistochemical results are positive, the molecular-based analysis is not necessary. However, based on intrinsic limitation of the sensitivity for the anti-E746-A750 antibody, a negative result with this IHC assay could not be used to exclude patients from molecular testing. For detection of exon 21 mutation status, molecular-based techniques are recommended to reduce the false negative rate.

In our meta-analysis, we have found that the mean DORs were 111.17 and 126.66 for the anti-E746-A750 antibody and the anti-L858R antibody, respectively, which have a high level of overall accuracy also.

Meta-analysis is a comprehensive method for analyzing multiple medical studies of the same type and purpose. Pooled data is a good option to use when overall included studies are homogeneous. Therefore, an exploration of the reasons for heterogeneity is an important goal of meta-analysis [19]. In present meta-analysis, an important method to detect heterogeneity was evaluated by the I^2^ test for the PDOR. Although we found statistically significant heterogeneity for sensitivity, specificity, PLR, and NLR for the anti-E746-A750 antibody, however, there were no heterogeneity between DORs, heterogeneity chi-squared  = 16.02 (p = 0.3124) and I^2^ = 12.6%. For anti-L858R antibody, we noticed statistically substantial heterogeneity for DOR, heterogeneity chi-squared  = 38.70 (p = 0.0002) and I^2^ = 66.4%. In order to explore the potential source of heterogeneity, we performed meta-regression and sub-group analysis. We did not observe heterogeneity between the higher quality (STARD score ≥13; QUADAS score ≥10) and the lower quality studies. Differences among studies with or without blinded design and IHC methodology (TMA vs. individual slides) did not reach statistical significance. However, we noted the differences among studies about consecutive or random design (p = 0.0333), IHC score criteria (consider 2+ or 3+ as positive vs. others) (p = 0.0262), and standard (direct sequencing vs. others) (p = 0.0102) had statistical significance. This finding implies that consecutive or random design, IHC score criteria, and standard had substantially impact on the diagnostic accuracy. In sub-group analysis, based on these three sources of heterogeneity, we set up six subgroups for E740-A750 and L858R respectively to further explore the heterogeneity. For studies in 2+ or 3+ staining as positive sub-group for L858R, we observed the numbers of sensitivity, PLR, NLR, and DOR of sub-group significantly surpassed the others sub-group with significantly decreased consistency coefficient, as shown in Table 6. Therefore, we strongly recommend using the 4 grades visual score criteria (consider 2+ or 3+ as positive) as the standard IHC staining scoring criteria to detect EGFR mutation status in NSCLC. Moreover, different standard could also affect the consistency. Detection by Direct sequencing method for E746-A750 showed more homogeneous than non-Direct sequencing method (Table 5). With uniform standard and IHC score criteria, we can not only reduce the difference among different readers, but also improve the diagnostic value of mutation-specific antibodies for detection of EGFR mutations in NSCLC.

In developing countries, especially in undeveloped areas, high-tech molecular-based detection techniques are difficult to access. However, IHC is cost-effective and widely-available, which can be performed on a large scale. In addition, IHC is easy to perform and not time-intensive, making it popular with both clinicians and technicians. Moreover, IHC can provide reliable results with only a limited amount of tissue material, such as small biopsies or cytological samples. However, there are some limitations to the mutation-specific antibodies. Currently, the availability of only two antibodies would be considered insufficient for clinical application, as rarer, sensitizing EGFR mutations could not be detected. Additionally, considering a variety of IHC staining criteria were used in the studies, it would be necessary to establish a uniform immunohistochemical staining protocol.

In conclusion, we recommend using the immunohistochemical method alone for detection of NSCLC EGFR mutation if results are positive for EGFR mutation status. If detection of mutations in exon 19 have a negative result after IHC, molecular-based analyses become necessary. However, for exon 21 mutations, we recommend using confirmatory molecular testing if time and economic resources permit. In summary, mutation-specific antibodies for immunohistochemical detection of EGFR mutation status is a novel cost-effective [9], and widely-available method that deserves further investigation.

## Supporting Information

Checklist S1 **PRISMA checklist.**
(DOC)
